# Impact of obesity and type 2 diabetes on muscle power, quality, and force–velocity, and their relation to functional capacity

**DOI:** 10.1007/s40200-026-01995-4

**Published:** 2026-07-03

**Authors:** Anders Stouge, Anders Hammer Nielsen-Kudsk, Michael Vaeggemose, Hatice Tankisi, Jens Meldgaard Bruun, Henning Andersen

**Affiliations:** 1 Department of Neurology, Aarhus University Hospital, Aarhus, Denmark; 2 Steno Diabetes Center Aarhus, Aarhus, Denmark; 3 The MR Research Centre, Aarhus University, Aarhus, Denmark; 4 Department of Clinical Medicine, Aarhus University, Aarhus, Denmark; 5 GE HealthCare, Brøndby, Denmark; 6 Department of Neurophysiology, Aarhus University Hospital, Aarhus, Denmark

**Keywords:** Type 2 diabetes, Severe obesity, Diabetic myopathy, Muscle failure, Functional impairment, Neuromuscular dysfunction

## Abstract

**Background:**

Obesity and type 2 diabetes (T2D) increase the risk of sarcopenia and mobility decline, yet the underlying muscle contractile alterations remain poorly understood. This study investigated how severe obesity and T2D affect muscle power, force–velocity relationships, and muscle quality.

**Methods:**

In this cross-sectional study, 45 middle-aged individuals were categorized as non-obesity (Non-O; BMI 18.5–30 kg/m^2^), obesity (O; BMI ≥ 35 kg/m^2^), and obesity with T2D (O + T2D; BMI ≥ 35 kg/m^2^). Isokinetic torque and power of knee extensors (KE) and dorsiflexors (DF) were measured (DF: 0–120°/s; KE: 0–270°/s). Muscle volume and fat infiltration (FF, %) were quantified using MRI. Outcomes included absolute, specific (relative to muscle volume), and normalized (relative to body weight) power. Functional capacity was assessed with five-times sit-to-stand (5xSTS) and 10-m walk (10MWT) tests.

**Results:**

KE power was 51W lower in O + T2D than O (P = 0.008) with larger deficits at higher velocities (interaction, P = 0.027). O and O + T2D exhibited lower normalized KE power (–0.8 and –1.1 W/kg vs. Non-O; both P < 0.001). KE FF was higher in O (5%) than Non-O (3%, P = 0.003), and highest in O + T2D (7%, P = 0.023). DF torque declined faster with velocity in O and O + T2D (P ≤ 0.012). Specific power did not differ. KE normalized power was the strongest predictor of performance (5xSTS: R^2^ = 0.57,P = 0.003; 10MWT: R^2^ = 0.71,P < 0.001).

**Conclusions:**

Severe obesity impairs normalized muscle power, with T2D exacerbating KE power deficits and fatty infiltration. These muscle contractile impairments may contribute to functional decline already in middle-aged individuals.

## Introduction

Ageing is characterized by a progressive decline in muscle strength, mass, and function, which is further accelerated by chronic diseases such as type 2 diabetes (T2D) [1]. Compared to normoglycemic individuals, those with T2D face an increased risk of falls, sarcopenia, and frailty [2, 3], adversely affecting quality of life and overall morbidity and mortality [4, 5]. Promoting healthy ageing by maintaining functional capacity and independence in at-risk populations has become a public health priority [6–8]. Therefore, advancing our understanding and characterization of muscle deterioration in T2D, particularly in middle-aged individuals before overt mobility limitations emerge, is important for identifying targets for early intervention.

Insulin resistance, chronic hyperglycemia, and other features of the metabolic syndrome have alongside diabetes-related polyneuropathy (DPN) been linked to sarcopenia and functional decline [9–12]. Obesity, which is closely associated with T2D, is also an independent risk factor for functional impairment [13–17]. Although DPN is known to cause motor deficits and atrophy [18, 19], its limited contribution to sarcopenia in the general T2D population [10] suggests that other drivers of muscle contractile dysfunction in T2D remain to be explored. However, muscle contractile function in individuals with obesity and T2D in the absence of DPN has not been well characterized.

While obesity promotes muscle strength and mass, muscle quality and function may suffer, reflecting a paradox where functional capacity declines despite strength gains [20]. Accordingly, the ratio of muscle strength to adipose tissue mass, rather than absolute strength alone may hold greater clinical implications [21, 22]. Notably, with ageing, muscle power (strength × velocity) declines earlier and more sharply than strength or mass alone [23, 24]. This disproportionate decline in power relative to mass indicates that intrinsic factors, collectively referred to as muscle quality [23], may drive early functional impairments. Key indicators of muscle quality include specific strength or power (torque or power normalized to muscle volume or mass) [25, 26], and myosteatosis, defined as fat infiltration within skeletal muscles [21]. Thus, assessing these parameters may provide additional insights into muscle dysfunction. Importantly, several intramuscular mechanisms have been proposed to impair contractile function in T2D, including altered calcium dynamics [27, 28], impaired cross-bridge kinetics [21], and mitochondrial dysfunction [29], with type II fibers appearing particularly vulnerable [21, 30]. As these processes are critical for rapid force development, their disruption may disproportionately impair contraction velocity, and consequently, velocity-dependent muscle power, rather than strength, similar to patterns observed with ageing.

In T2D, few studies have investigated muscle power and these studies have focused on patients aged > 60 years with DPN. Therefore, the effects of T2D per se remain unclear [31–34]. Similarly, while obesity has been linked to preserved or increased muscle power, body weight-normalized power is often reduced [17, 35]. However, most studies have relied on indirect functional tests (e.g., chair stand and jump tests) rather than direct assessments of power, warranting further investigation. These findings underscore the need to assess muscle power and quality, especially in middle-aged adults who may still retain normal muscle strength and mass.

To address these gaps, we aimed to assess skeletal muscle contractile function beyond measures of strength and size. Specifically, we evaluated muscle quality, power, and force–velocity characteristics and their relationships with functional capacity in middle-aged individuals with class II and III obesity (BMI ≥ 35 kg/m^2^) with and without T2D. We hypothesized that while obesity would be associated with similar or greater absolute strength and power compared to controls without obesity, the addition of T2D would result in impaired muscle power and quality, with a disproportionate impairment at higher contraction velocities. Furthermore, we hypothesized that power normalized to body weight would correlate more strongly with functional capacity than absolute strength.

## Methods

### Study design and participants

This cross-sectional study included adults with class II and III obesity (BMI ≥ 35 kg/m^2^) with or without T2D recruited between December 2022 and December 2024 from referrals for assisted weight loss or diabetes management at Steno Diabetes Center Aarhus and the Regional Hospital of Viborg, Denmark. Additionally, participants were recruited through advertisements on social media. Inclusion criteria were age between 18–60 years, BMI ≥ 35 kg/m^2^, and self-reported activity level of three hours per week or less, indicating a sedentary to recreationally active lifestyle. Participants with obesity were stratified into those without (O) and with T2D (O + T2D).

Individuals without obesity (Non-O) were recruited as relatives of participants or via social media. Inclusion criteria included BMI ≥ 18.5 and < 30 kg/m^2^ and a self-reported sedentary to recreationally active lifestyle as described for participants with obesity. Matching was done by age (± 5 years), sex, height (± 10 cm), and weight (obesity groups only; ± 10 kg). Exclusion criteria included polyneuropathy, uncontrolled cardiopulmonary disease, neuromuscular disorders, acute or unstable endocrine disorders, imaging-confirmed arthrosis affecting daily life function, significant peripheral artery disease (PAD), or any magnetic resonance imaging (MRI) contraindications. Non-O participants with prediabetes (HbA1c ≥ 42 mmol/mol) were excluded. All participants gave written informed consent. Ethical approval was obtained from the Danish Research Ethics Committees (1–10–72–385-21). The study was conducted in accordance with the Declaration of Helsinki, and was preregistered at ClinicalTrials.gov (NCT05685927).

### Screening for neuropathy and peripheral artery disease

All participants were screened for DPN and PAD according to the study’s exclusion criteria. Polyneuropathy exam included clinical assessments and nerve conduction studies (NCS). DPN was defined according to the Toronto criteria: symptoms and/or signs of neuropathy combined with abnormal NCS in ≥ 2 nerves, including the sural nerve [36, 37]. A Toronto Clinical Neuropathy Score (TCNS) > 5 was considered suggestive of DPN [38]. PAD was defined as an ankle-brachial index (ABI; ratio of systolic blood pressure at the ankle to that in the arm) < 0.9.

### Clinical profiling

Fasting blood samples were collected prior to intake of any diabetes-related medications, followed by a weight-adjusted standard breakfast (low-carb energy bar, drink, crispbread, fruit). Analyses included HbA1c, plasma glucose, insulin, creatine kinase, and high-sensitivity CRP. Insulin resistance was estimated using the HOMA2 calculator (Diabetes Trials Unit, University of Oxford, UK) [39]. Additional blood parameters (i.e. total cholesterol, LDL, HDL, and triglycerides) were retrieved from recent clinical records (within 12 months of study enrollment).

Anthropometric measurements included height, weight, waist circumference, thigh and lower leg lengths, with limb segment lengths used for MRI setup.

### Daily activity and fall risk stratification

Physical activity was measured using a thigh-worn accelerometer (ActiGraph GT3X +, Pensacola, FL, USA). ActiLife software was used to calculate total and moderate-to-vigorous physical activity (MVPA) levels [40]. The accelerometer was worn continuously between the dynamometry session and MRI session, with valid data requiring at least three days of recording. Participants were asked about falls and fear of falling consistent with recommended guidelines for fall risk stratification [41].

### Dynamometry

All participants underwent isometric and isokinetic muscle testing of the knee extensors (KE) and ankle dorsiflexors (DF) on their non-dominant leg using a Biodex System 3 Pro dynamometer (Biodex Medical Systems, Shirley, NY, USA). Testing order was standardized: KE preceded DF, and isokinetic testing was performed before isometric testing. Participants performed 20 submaximal contractions for KE and DF at various isokinetic velocities for warm-up and familiarization. Valid trials required a coefficient of variation (CV) for torque values ≤ 10%, following previous protocols [42, 43].

Knee extension testing. Participants were seated with their hip fixed at 70° flexion, and the dynamometer arm secured to the lower leg just above the medial malleolus. The range of motion (ROM) was limited to 80° (from 90° knee flexion to 10° knee flexion). Isokinetic contractions were performed at 90°, 180°, and 270^o^/s. Eight maximal concentric contractions were performed at each velocity, with 15-s rest intervals between contractions and 2-min rest periods between velocity conditions. Maximal voluntary isometric contraction (MVIC) was tested at 90° knee flexion, with three trials, each separated by 45 s. A 10-min break followed isokinetic testing before MVIC assessment.

Ankle dorsiflexion testing. Participants were seated with the hip at 40° flexion and knee at 160^O^ extension, with the foot secured to the footplate. ROM was limited to 30 (from 30^0^ to 0° plantarflexion), ensuring all participants could comfortably generate torque throughout the range. Isokinetic contractions were performed at 30°, 60°, and 120°/s, and MVIC was measured at 30^o^ following the same procedure as for knee extensor testing.

Isokinetic velocities, for both KE and DF, were chosen based on evidence of feasible velocities in healthy young and older adults [44].

Muscle power evaluation. Muscle power was derived from the torque and velocity data for each velocity condition sampled at 100Hz. The peak contraction at each angular velocity was identified and the area under the curve (AUC) was computed using at custom MATLAB script (MATLAB R2022b, MathWorks, Natick, MA, USA). AUC was used to calculate the average power to evaluate power consistency throughout the peak contraction. Moreover, for both KE and DF testing, velocity order of isokinetic contractions was randomized, and after completing all conditions, participants performed four additional contractions at the first tested velocity to assess fatigue and reliability of the isokinetic protocol.

### Functional capacity

At the end of the dynamometry session, participants performed the five times sit-to-stand (5xSTS) test and the 10-m walk test (10MWT). The 5xSTS measured the time to complete five repetitions, with the average of two trials recorded [45]. For the 10MWT, participants walked at maximally safe speed. Time was recorded over the central 6 m, and the average of three trials was used [24].

### Muscle volume, fat infiltration, and specific power

Muscle volume, fatty infiltration, and specific power were assessed by MRI. MRI was conducted 3–7 days after the dynamometry session using a 3 T whole-body scanner (MR750, GE HealthCare, WI, USA) and a 32-channel Body Array Coil. Participants were positioned supine, feet-first, and the body-coil was placed around the thighs and lower legs. The field of view (480 × 276 × 186 mm) was centered based on measured thigh and lower leg lengths. Imaging sequence and segmentation was adapted from previously established procedures in T2D [46]. A 3D proton density-weighted Dixon based IDEAL-IQ sequence was used for fat fraction mapping, Imaging parameters were: repetition time = 7.9 ms, echo time = [1–6] msec, number of echoes = 6, flip angle = 4°, voxel size = 1.5 × 1.5 × 6.0 mm, bandwidth = 111.11 kHz, acceleration factor = 2, no interslice gap, and scan time of 2 min and 8 s. Muscle volumes of KE and DF were estimated in ITK-SNAP (v4.2.2; http://www.itksnap.org). Segmentation was conducted by a single blinded examiner (AHNK), and images were reviewed for artifacts and distortions (AS). Fat infiltration was expressed as a fat fraction (FF, %), and contractile muscle volume (CMV) was calculated by adjusting total volume for FF. Specific power was defined as average muscle power normalized to CMV (W/kg).

### Statistical analysis

All analyses were performed using STATA 16 (StataCorp LLC, TX, USA). Data normality was assessed via Q–Q plots and histograms. A priori sample size estimation for the primary outcome (muscle power) was based on an expected effect size of 1.1 for comparisons between groups with and without T2D (α = 0.05, power = 0.80), requiring at least 15 participants per group (G*Power 3.1.9.4, Universität Kiel, Germany) [33, 47].

Between-group differences in CMV, FF, and baseline variables (i.e., physical activity, functional capacity and hs-CRP) were assessed using two-way ANOVA (group × sex). A three-way mixed model (group × sex × velocity) was used to evaluate interactions across velocity conditions for force, power, specific power, and normalized power, as well as main group effects independent of velocity. Subject ID was included as a random effect and velocity was treated as a categorical variable. Analyses were not stratified by sex, as no significant interactions were observed and stratification would reduce statistical power. Post hoc comparisons were performed using Tukey’s HSD or the Benjamini–Hochberg method, depending on data characteristics.

Linear regression models were used to assess associations between muscle function and functional capacity. First, univariate analyses were performed for each muscle functional domain. Variables were subsequently entered into multivariate linear regression models to identify independent associations with functional performance outcomes. Model assumptions including linearity, residual distribution and homoscedasticity were verified. Multicollinearity was defined using the variance inflation factors (VIF), and variables with VIF > 5 were excluded. Model fit was evaluated with R^2^. Reliability of KE and DF protocols was assessed using intraclass correlation coefficients (ICC).

Analyses were conducted using complete-case data. Data are reported as mean ± standard deviation (SD) or mean [95% Confidence Interval] (CI). Statistical significance was set at p < 0.05.

## Results

### Participants characteristics

**Fig. 1:**
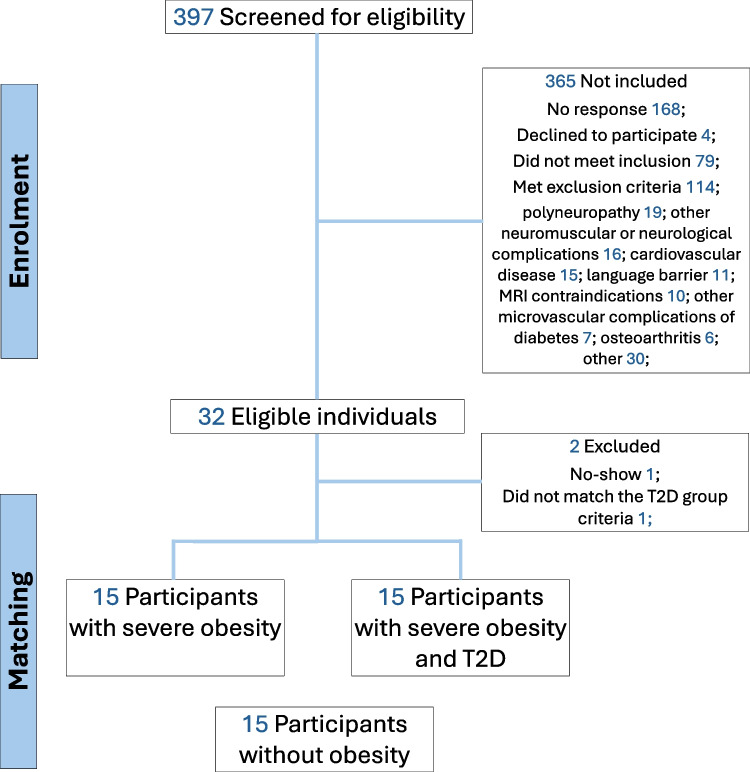
Flowchart illustrating participant enrolment, eligibility screening, inclusion and exclusion process, and final group assignment. Matching was based on age, sex, and height, with additional body weight matching for obesity groups. T2D = Type 2 diabetes

As outlined in Fig. 1, 397 individuals with obesity were screened, with 32 being eligible and 30 included.

Participant characteristics are presented in Table 1. Groups were comparable concerning TCNS scores, NCS parameters, PA levels, ABI, and creatine kinase levels. Compared to Non-O, participants with obesity had higher HOMA-IR values, elevated markers of low-grade inflammation (hs-CRP), slower gait speed, and took longer to complete the 5xSTS. Participants with T2D engaged less in MVPA than Non-O and differed from O participants in diabetes-related parameters and medication. The frequency of reported falls and fear of falling did not differ between groups. Missing data included: MRI (n = 3, O + T2D), 10MWT (n = 1, O + T2D), and NCS data due to technical issues or refusal (various nerves: Non-O = 2; O = 1; O + T2D = 4).

**Table 1:** Baseline characteristics and matching criteria

Characteristics	Non-O	O	O + T2D
N	15	15	15
Age, years	48 ± 10	46 ± 7	48 ± 7
Sex (female), N (%)	9 (60)	10 (67)	9 (60)
BMI, kg/m^2^	24 ± 3	40 ± 3	42 ± 6
Weight, kg	73 ± 14	118 ± 10	120 ± 25
Height, cm	174 ± 9	172 ± 7	168 ± 10
Waist, cm	84 ± 11	120 ± 11	126 ± 16
**Diabetes profile**			
Diabetes duration, years	-	-	7 ± 7
HbA1c, mmol/mol	34 ± 3	35 ± 3	52 ± 17
F-glucose, mmol/l	5.3 ± 0.3	5.6 ± 0.5	8.3 ± 2.8
F-Insulin, pmol/l	48 ± 21	113 ± 51	164 ± 67
HOMA-IR, a.u	0.9 ± 0.4	2.1 ± 1.0	3.3 ± 1.3
**Neuropathy assessment**			
TCNS	3 ± 1	3 ± 2	4 ± 2
Sur dist SNAP, μV	5 ± 2	3 ± 2	4 ± 2
Sur NCV, m/s	46 ± 7	48 ± 6	53 ± 8
Per dist CMAP, μV	8 ± 2	8 ± 2	9 ± 2
Per NCV, m/s	48 ± 3	47 ± 3	48 ± 6
**Physical activity profile**			
Physical activity, (min/day)	159 ± 37	138 ± 34	124 ± 46
Moderate-to-vigorous physical activity^**a**^,(min/day)	45 ± 11	32 ± 10	25 ± 12
**Functional capacity**			
Five time sit-to-stand^**a,c**^, s	7.1 ± 1.5	10.0 ± 1.3	10.4 ± 2.3
Gait speed^**a,c**^, m/s	1.98 ± 0.18	1.51 ± 0.18	1.39 ± 0.11
**Fall risk stratification**			
Any falls in the past year, N (%)	2 (13)	5 (33)	7 (47)
Fear of falling, N (%)	2 (13)	3 (20)	4 (27)
**PAD screening**			
Ankle-Brachial index	1.25 ± 0.10	1.20 ± 0.12	1.20 ± 0.13
**Medication**			
Insulin, N (%)	-	-	3 (20)
oral anti-diabetic drugs, N (%)	-	-	15 (100)
Antihypertensives, N (%)	1 (7)	7 (47)	8 (53)
Statins, N (%)	0 (0)	1 (7)	12 (80)
**Inflammation and muscle panel**			
hs-CRP^**a,c**^, mg/l	1.1 ± 1.3	4.3 ± 4.2	5.6 ± 4.2
Creatine kinase, (U/l)	129 ± 71	118 ± 87	96 ± 52
**Lipid profile**			
Total cholesterol, mmol/l	4.9 ± 0.5	4.7 ± 0.8	3.9 ± 0.9
LDL-cholesterol, mmol/l	2.9 ± 0.7	2.7 ± 0.5	1.9 ± 0.7
HDL-cholesterol, mmol/l	1.7 ± 0.3	1.2 ± 0.3	1.0 ± 0.2
Triglycerides, mmol/l	0.8 ± 0.2	1.9 ± 0.7	3.4 ± 3.6

Values are reported as mean ± SD or number (%). Group labels: Non-O = individuals without obesity; O = individuals with obesity; O + T2D = individuals with obesity and type 2 diabetes. TCNS = Toronto Clinical Neuropathy Scoring system. Sur dist SNAP = sensory nerve action potential of the distal sural nerve. Sur NCV = sural nerve conduction velocity. Per dist CMAP = compound muscle action potential of the distal peroneal nerve. Per NCV = peroneal nerve conduction velocity. a.u. = arbitrary unit

Post hoc comparisons (Tukey’s HSD):

**a** = O + T2D significantly different from Non-O

**b** = O + T2D significantly different from O

**c** = O significantly different from Non-O

### Muscle volume and fat infiltration

**Fig. 2:**
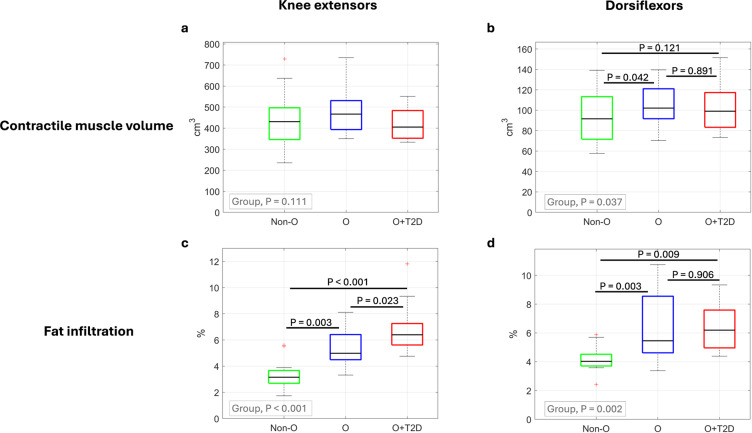
Box plot of contractile muscle volume (CMV) and fatty infiltration, expressed as fat fraction (FF%). (a) CMV of the knee extensors. (b) CMV of the dorsiflexors. (c) FF of the knee extensors. (d) FF of the dorsiflexors. Data are presented as the median ± interquartile range (IQR), with whiskers extending to the minimum and maximum values within 1.5 × IQR. Outliers are indicated by " + ". Non-O = Non-obesity, O = Obesity, O + T2D = Obesity with type 2 diabetes. P-values presented are for the main effect of groups, and if relevant, followed by Tukey’s HSD for post-hoc analyses of between group differences. Relevant sex-specific differences for these parameters are presented in the results section

Figure 2 presents box plots of CMV and FF for the KE and DF. Fat infiltration (%) was significantly higher in O as compared to Non-O for both muscle groups (P = 0.003): KE: 5.4 ± 1.5 vs. 3.3 ± 1.1, DF: 6.5 ± 2.4 vs. 4.2 ± 0.9. KE fat infiltration was even greater in O + T2D than O (6.9 ± 2.0 vs. 5.4 ± 1.5, P = 0.023). DF fat infiltration did not differ significantly within groups with obesity (6.4 ± 1.5 vs. 6.5 ± 2.4, P = 0.906).

CMV was largely similar between groups. O had slightly greater DF CMV (cm^3^) than Non-O (105 ± 19 vs. 93 ± 25, P = 0.042). No significant differences were seen between O + T2D and the other groups.

### Torque-velocity relationship

**Fig. 3:**
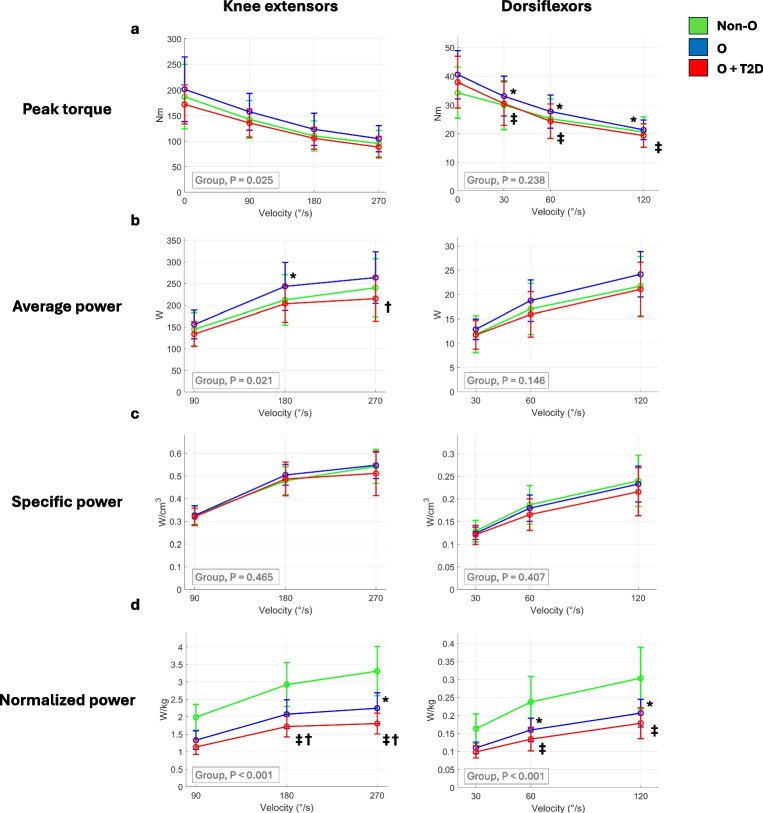
Force- and power-velocity curves for the knee extensors (KE) and dorsiflexors (DF). (a) Peak torque of KE and DF across increasing velocities (Nm). (b) Average power of the peak contraction for KE and DF (W). (c) Specific power, defined as average power normalized to contractile muscle volume (W/cm^3^). (d) Normalized power, defined as average power normalized to body weight (W/kg). Data are presented as mean ± SD. P-values reflect main group effects. Significant group × velocity interactions were further examined using Benjamini–Hochberg-adjusted post hoc tests and are indicated as follows: ‡ O + T2D compared to Non-O. † O + T2D compared to O. * O compared to Non-O. Group abbreviations: Non-O = Non-obesity, O = Obesity, O + T2D = Obesity with type 2 diabetes

Figure 3a illustrates KE and DF force–velocity curves. In DF, peak torque did not differ across groups (main group effect: P = 0.238), but torque decreased more with increasing velocity in O and O + T2D. From MVIC to 120°/s torque declined by 46.6 ± 7.0% in O vs. 39.5 ± 8.7% in Non-O (P = 0.012). No difference in torque decline was observed between O + T2D and O (48.2 ± 8.2% vs. 46.6 ± 7.0%, P = 0.642).

In KE, peak torque output differed between groups (main group effect: P = 0.025). O torque was higher than O + T2D across all velocities (mean difference: 18 Nm; 95% CI [4, 32]; P = 0.012). No significant differences were seen between O vs. Non-O (mean difference: 11 Nm; 95% CI [−2; 24]; P = 0.088) or between O + T2D and Non-O (mean difference: −7 Nm; 95% CI [−20; 6]; P = 0.298). No group differences were observed in the force–velocity slope.

### Power-velocity relationship

O + T2D showed a blunted increase in KE power with increasing contraction velocities when compared to O (90°/s to 270°/s, –27 W; 95% CI [–50, –3]; *P* = 0.027) (Fig. 3b). DF power showed a similar pattern but was not statistically significant (30°/s to 120°/s, –1.9 W; 95% CI [–4.3, 0.5]; *P* = 0.118). Across all velocities, KE power was lower in O + T2D vs. O (mean difference: –51 W; 95% CI [–88, –14]; *P* = 0.008).

Compared to Non-O participants, O showed a greater KE power increase between 90°/s and 180°/s (difference: 20 W; 95% CI [2, 38]; *P* = 0.028). No group differences were found in the power–velocity slope for KE or DF between O + T2D and Non-O.

When muscle power was normalized to body weight, both O and O + T2D had lower power than Non-O across all velocities (Fig. 3d). *DF*: O vs. Non-O; –0.10 W/kg; 95% CI [–0.14, –0.05] (P < 0.001), and O + T2D vs. Non-O; –0.13 W/kg; 95% CI [–0.17, –0.08] (P < 0.001). *KE*: O vs. Non-O; –0.8 W/kg; 95% CI [–1.1, –0.4] (P < 0.001), and O + T2D vs. Non-O; –1.1 W/kg; 95% CI [–1.4, –0.8] (P < 0.001). The slope of normalized power with velocity also increased less in O and O + T2D vs. Non-O. For DF (30°/s vs. 120°/s), the deficit was –0.04 W/kg in O (95% CI [–0.08, –0.01]; P = 0.007) and –0.06 W/kg in O + T2D (95% CI [–0.09, –0.03]; P < 0.001). For KE (90°/s vs. 270°/s), deficits were –0.4 W/kg in O (95% CI [–0.6, –0.1]; P = 0.001) and –0.7 W/kg in O + T2D (95% CI [–0.9, –0.4]; P < 0.001). Notably, the slope of KE was even more impaired in O + T2D vs. O (–0.3 W/kg; 95% CI [–0.5, –0.2]; P < 0.001).

When scaled to CMV, no significant differences were observed in overall effect or slopes of the specific power-velocity relationship (Fig. 3c).

### Reliability of dynamometry tests and sex effects

Test–retest reliability was high and comparable across groups for both KE and DF (ICC range 0.94–0.99, P = 0.368) (detailed data not shown), indicating fatigue did not affect performance. Females had lower muscle volume, as well as lower peak torque and average power across all velocities for both KE and DF. No sex × velocity interactions were found.

### Relationship between functional capacity and muscle functional domains

A multivariate linear regression analysis was performed to examine how well muscle functional domains (peak torque, normalized power, and fat infiltration) explain performance in functional capacity tests (5xSTS and 10MWT) (see Table 2).

**Table 2:** Univariate and multivariate linear regression analysis of muscle functional domains and functional performance

	**Univariate**			Multivariate*			
	**β**	**P**	**R**^**2**^	**β**	**P**	**R**^**2**^	
**5xSTS (s)**							
**Peak torque, KE**	0.01	0.56	0.01	0.02	0.09	0.57	
**Peak torque, DF**	−0.1	0.91	0.00	-	-		
**Normalized power, KE**	−1.7	** < 0.001**	0.36	−1.6	**0.003**		
**Normalized power, DF**	−16.7	** < 0.001**	0.35	-	-		
**Fat fraction, KE**	0.6	** < 0.001**	0.38	0.3	0.13		
**10MWT (m/s)**							
**Peak torque, KE**	0.002	0.21	0.04	-	-	0.71	
**Peak torque, DF**	0.01	0.32	0.02	−0.01	0.35		
**Normalized power, KE**	0.3	** < 0.001**	0.64	0.22	** < 0.001**		
**Normalized power, DF**	2.6	** < 0.001**	0.46	-	-		
**Fat fraction, KE**	−0.1	** < 0.001**	0.43	−0.02	0.30		

Univariate and multivariate linear regression analyses examining associations between muscle functional domains and functional performance. Multivariate models included variables from the different muscle domains entered simultaneously and were adjusted for moderate-to-vigorous physical activity. KE = knee extensors. DF = dorsiflexors. Peak torques refer to the maximum torque for KE and DF at their fastest isokinetic velocity (Nm). Normalized power = average power of the peak torque contraction normalized to body weight (W/kg). Fat fraction refers to the fatty infiltration of KE as a percentage (%). Parameters demonstrating multicollinearity were not included in the multivariate model, as indicated by ‘-’. β = standard beta coefficient. 5xSTS = five-time sit-to-stand. 10MWT = 10-m walk test

^*^the multivariate model was adjusted for moderate-to-vigorous physical activity level. Its effect was not statistically significant in the multivariate model (5xSTS: β: 0.02, 95% CI [−0.02;0.07], P = 0.30) and 10MWT: β: 0.004, 95% CI [−0.002;0.010], P = 0.15)

The model showed that 5xSTS performance was partly explained by knee extensor peak torque, normalized power, and fat infiltration (R^2^ = 0.57). Only normalized power contributed significantly to the model (β: −2, 95% CI [−3;−1]; P = 0.003), even after adjusting for MVPA.

For 10MWT, gait speed was partly explained by dorsiflexor peak torque, knee extensor normalized power, and knee extensor fat infiltration (R^2^ = 0.71). Similarly, only normalized power was a significant contributor (β: 0.2 95% CI [0.1;0.3]; P < 0.001), despite adjusting for MVPA.

## Discussion

This study provides a detailed dynamometric assessment of multiple muscle contractile domains in two locomotor muscles of individuals with severe obesity, with and without T2D. A major strength of the study is the careful matching of groups and the comprehensive design, allowing us to isolate the added impact of T2D, independent of DPN, on muscle function in severe obesity. To date, this has been only sparsely investigated in this population. The key finding is a reduced ability of individuals with severe obesity and T2D to scale muscle power with increasing contraction velocity, particularly in KE. This indicates a diminished functional reserve, whereby the capacity to generate power under high-velocity conditions is impaired, despite preserved muscle strength and mass.

Consistently, deficits in both KE absolute and body weight–normalized power were more evident at higher contraction speeds, alongside greater fat infiltration in T2D compared to obesity alone. Moreover, normalized power deficits across all velocities were more pronounced in both KE and DF among groups with obesity, with and without T2D, compared to individuals without obesity. Importantly, normalized power emerged as the strongest predictor of physical performance (i.e., 5xSTS and 10MWT), even after adjusting for physical activity. These findings demonstrate that severe obesity combined with T2D impair muscle contractile function, beyond strength and size, highlighting the relevance of assessing muscle power alongside strength in middle-aged individuals and of expressing power relative to body weight for functional interpretation.

### Impact of severe obesity on muscle function

Individuals with severe obesity without T2D demonstrated preserved or greater contractile strength and muscle volume in both KE and DF compared to those with T2D and individuals without obesity. These findings align with prior evidence suggesting that obesity may confer a protective effect on absolute muscle mass and strength, particularly in weight-bearing muscles such as the KE [47–49]. Similar effects have also been reported in the DF [50, 51].

Specific muscle power was preserved in obesity, though prior studies have reported conflicting results. Some studies report no change in torque or power normalized to muscle volume [35, 47, 52], while others describe reduced specific strength in this population [17, 48, 53]. These discrepancies are likely due to methodological differences in muscle volume assessment. Studies using bioimpedance or DEXA often report reduced specific strength without adequately accounting for myosteatosis [17, 48, 53]. In contrast, our study used MRI, providing more precise estimates of muscle volume and fat infiltration.

Across all contraction velocities, absolute power was preserved in both KE and DF, with a tendency toward greater KE power in individuals with obesity, indicating no impairment in sustaining absolute power production. While one study has examined muscle power using isokinetic dynamometry [47], others have derived power from jump tests [35] or examined hypergravity-trained athletes rather than directly assessing individuals with obesity [54]. Despite methodological differences, our results align with prior evidence suggesting that obesity does not compromise absolute muscle power and may even be associated with increased power output. However, this does not necessarily translate into functional benefits, as the excess mechanical and metabolic demands of obesity must be considered. Supporting this notion, individuals with obesity exhibited lower functional capacity in the 5xSTS and 10MWT, despite preserved absolute power. In contrast, muscle power normalized to body weight was significantly reduced in individuals with obesity for both KE and DF, consistent with studies showing that excess adiposity impairs normalized strength and power, even when absolute levels are maintained or enhanced [20].

### Impact of T2D on muscle function

Compared to individuals with severe obesity alone, those with T2D exhibited additional impairments in muscle quality and function. Despite similar muscle volume, MRI revealed greater fatty infiltration in knee extensors of the T2D group, supporting the role of ectopic fat accumulation in muscle dysfunction. This finding aligns with frameworks to redefine obesity classification by incorporating measures of adiposity beyond BMI [55]. Fat infiltration, and associated lipotoxicity are linked to hyperglycemia, insulin resistance, dyslipidemia, and systemic inflammation [21, 56, 57], all of which were more pronounced in our T2D group.

Interestingly, specific power did not differ between groups when accounting for fat infiltration. This contrasts with population-based studies, where specific strength tends to decline more significantly than absolute strength and muscle volume in T2D cohorts [11, 12, 25, 26]. This discrepancy may partly reflect methodological differences, as discussed above, with our study providing a more detailed evaluation of specific power. Another explanation is that our T2D participants had well-controlled diabetes, as previous studies have suggested a stronger association between elevated HbA1c levels and reduced specific strength [11, 12]. Diminished specific strength may also reflect neuropathy-related impairments rather than T2D per se, as it has predominantly been reported in individuals with DPN [18, 28, 34, 46, 58]. Our detailed neuropathy screening rules out any contribution to the deficits by DPN. Moreover, normalization to contractile muscle volume primarily reflects macroscopic muscle properties and may not adequately capture intracellular or metabolic impairments. Consequently, specific power does not fully represent muscle quality in T2D, where intramuscular alterations associated with insulin resistance, such as mitochondrial dysfunction and intramyocellular lipid accumulation, are well described [29, 59], and may already present with obesity per se [60].

Beyond alterations in muscle composition, individuals with T2D exhibited a greater decline in both absolute and normalized KE power at higher contraction velocities, compared to those with obesity alone, and lower normalized KE and DF power than individuals without obesity. This suggests that T2D disrupts the power-velocity relationship, with minimal power advantages when transitioning from moderate to fast contraction speeds. This power plateau may partly explain their slower gait speed. In contrast, 5xSTS performance was comparable between obesity groups, consistent with findings that chair-stand performance plateaus at BMI levels above 30 kg/m^2^, which may reflect the more strength based nature of 5xSTS compared to gait speed [15]. Existing research on power output in T2D indicates lower absolute and specific power in DF, reduced normalized power at the knee, and a stronger association between power (rather than torque) and gait speed [31, 32, 34]. However, among these studies, only Volpato et al. accounted for neuropathy (regression analyses), leaving the direct effects of T2D on muscle power unclear. Our study addresses this gap by isolating the impact of T2D independent of neuropathy.

Overall, our findings suggest that muscle power deficits and fatty infiltration are exacerbated by the presence of T2D and its associated metabolic disturbances. This supports the notion that hyperglycemia, inflammation, and metabolic syndrome, rather than BMI, determine whether obesity preserves or harm functional muscle health [9, 21, 61].

### Muscle-specific differences and underlying mechanisms

Comparisons between KE and DF revealed distinct patterns in muscle-specific impairments. KE power and quality were more affected by the presence of T2D, suggesting greater vulnerability to diabetes-related impairments. This aligns with the concept of accelerated muscle ageing in T2D, as KE function also tends to decline more than DF with ageing [44]. Despite this, KE force–velocity profiles remained unchanged, whereas DF exhibited a steeper decline at higher velocities in obesity, with no additional effect from T2D. Our findings contrast with those of Sacchetti et al. [33], who reported altered force–velocity curves in KE among sedentary and trained individuals with diabetes, with further decrements in the presence of DPN. However, their cohort included older adults with longer T2D duration and lower BMI, suggesting that KE impairments in force–velocity behavior may not manifest until later disease stages. Studies on DF force–velocity behavior in obesity and T2D are lacking, though slowed contractility has been reported in DF using twitch and voluntary force development tests [28, 62].

Several factors may explain the muscle-specific differences in function. KE, a large muscle group involved in high-intensity activities (e.g. sprinting and jumping), may be more susceptible to metabolic dysfunction. In contrast, DF are much smaller, richer in type I fibers, yet critical for gait [63, 64]. While KE could theoretically be affected by disuse due to their role in high-load movements, the relatively high physical activity levels in our cohort, where all groups met WHO physical activity recommendations, suggest that disuse alone is unlikely to explain the observed deficits. Moreover, the relatively high overall physical activity level in our cohort may have helped preserve DF function, buffering against the impact of T2D. Therefore, it is reasonable to conclude that physical activity differences had limited impact on muscle contractile function in our study. Furthermore, evaluating more sedentary individuals would likely disclose greater impairments across both KE and DF.

Mechanistically, obesity is associated with a shift toward fast-twitch glycolytic fibers, potentially enhancing strength and power, and contributing to the steeper force–velocity slope observed in DF [65]. In T2D, contractile function is thought to be impaired through multiple mechanisms including lipotoxicity [21], impaired insulin signaling [30], mitochondrial dysfunction [29], extracellular matrix disruption [66], and altered calcium handling [27], many of which disproportionately affect type II fibers [21, 30]. Given the knee extensors’ greater reliance on type II fibers, this muscle group may be particularly susceptible to contractile impairments. Moreover, the increased fat infiltration in KE observed in our study suggests the presence of greater metabolic strain and structural vulnerability compared to DF in T2D. Accordingly, KE appear especially vulnerable in individuals with T2D, helping to explain the more pronounced power deficits of KE observed in this group. Nevertheless, future studies linking specific intramuscular mechanisms with muscle functional deficits are warranted to further clarify the vulnerability of knee extensors in T2D.

Together these dual influences, obesity promoting force via fiber type shift, and T2D undermining contractility, may explain why obesity alone preserves muscle power, while its combination with T2D leads to power impairments.

### Correlations to physical performance and clinical implications

Regression analyses demonstrated that lower limb muscle function, particularly knee extensor normalized power, was significantly associated with physical performance in both the 5xSTS and 10MWT. These findings underscore the critical role of muscle power in mobility-related tasks, particularly relative to body weight, reinforcing evidence that power is a stronger determinant of functional performance than strength alone [32, 67]. Notably, the task-specific distinction between the 5xSTS and 10MWT, with the latter relying more on rapid force production and movement velocity, may explain the stronger association between normalized power and gait speed observed in our study. Importantly, this association persisted after adjusting for MVPA, suggesting that functional impairments in obesity and T2D are not solely due to activity levels.

These results highlight the importance of early detection of muscle deficits, potentially using simple functional tests, especially velocity-sensitive tests such as 10MWT, before overt mobility limitations occur. Moreover, given the higher risk of functional decline in this population, our findings support interventional strategies that specifically target muscle power, not just strength, in combination with weight loss and glycemic control, as these strategies may be crucial for preserving mobility and reducing disability risk in individuals with severe obesity and T2D [5].

### Limitations

Some limitations should be acknowledged. First, although groups were matched on age, sex, and height, one participant in the O group was unmatched on sex, which may have introduced minor variability. Second, despite similar self-reported activity levels at inclusion, differences in accelerometer-based MVPA levels persisted. Third, missing MRI and NCS data in a few individuals may have influenced group comparisons. Finally, the cross-sectional design impairs causal interpretation of the observed associations in muscle function and performance.

## Conclusions

This study examined locomotor muscle contractile function in individuals with severe obesity, with and without T2D, demonstrating that those with T2D exhibit more pronounced muscular deficits than individuals with severe obesity alone, even in the absence of DPN. Knee extensor muscles appeared particularly vulnerable to T2D-realted impairments, demonstrating greater fatty infiltration and reduced muscle power scaling with increasing contractile velocities. Notably, normalized knee extensor power emerged as a key determinant of physical performance, emphasizing the need for interventions that prioritize muscle power enhancement and support weight management to preserve mobility and functional independence.

These findings indicate that middle-aged individuals with severe obesity and T2D demonstrate functional decline and muscle contractile impairments, despite preserved strength and size, emphasizing the importance of assessing muscle power in this population. Future longitudinal studies are warranted to investigate the progression of these muscular impairments over time and the response to targeted interventions including exercise, weight-loss and improved glycemic control.

## Data Availability

The data that support the findings of this study are not openly available due to reasons of sensitivity and are available from the corresponding author upon reasonable request. Data are located in controlled access data storage at Aarhus University, Aarhus, Denmark.

## Abbreviations

ABI: Ankle-brachial index
CMV: Contractile muscle volume
DPN: Diabetic polyneuropathy
FF: Fat fraction
MRI: Magnetic Resonance Imaging
NCS: Nerve conduction studies
PAD: Peripheral artery disease
TCNS: Toronto Clinical Neuropathy Scoring system
T2D: Type 2 diabetes

## References

[1] de Souto Barreto P, Rolland Y, Ferrucci L, Arai H, Bischoff-Ferrari H, Duque G, et al. Looking at frailty and intrinsic capacity through a geroscience lens: the ICFSR & Geroscience Task Force. Nat Aging. 2023;3(12):1474–9. 10.1038/s43587-023-00531-w.37985720 10.1038/s43587-023-00531-wPMC12159420

[2] Sinclair AJ, Abdelhafiz AH. Unravelling the frailty syndrome in diabetes. Lancet Healthy Longev. 2021;2(11):e683–4. 10.1016/s2666-7568(21)00256-7.36098024 10.1016/S2666-7568(21)00256-7

[3] Izzo A, Massimino E, Riccardi G, Della Pepa G. A Narrative Review on Sarcopenia in Type 2 Diabetes Mellitus: Prevalence and Associated Factors. Nutrients. 2021;13(1):183. 10.3390/nu13010183.33435310 10.3390/nu13010183PMC7826709

[4] Liccini A, Malmstrom TK. Frailty and Sarcopenia as Predictors of Adverse Health Outcomes in Persons With Diabetes Mellitus. J Am Med Dir Assoc. 2016;17(9):846–51. 10.1016/j.jamda.2016.07.007.27569712 10.1016/j.jamda.2016.07.007

[5] Sinclair AJ, Abdelhafiz AH, Rodríguez-Mañas L. Frailty and sarcopenia - newly emerging and high impact complications of diabetes. J Diabetes Complications. 2017;31(9):1465–73. 10.1016/j.jdiacomp.2017.05.003.28669464 10.1016/j.jdiacomp.2017.05.003

[6] Wolfe RR. The underappreciated role of muscle in health and disease. Am J Clin Nutr. 2006;84(3):475–82. 10.1093/ajcn/84.3.475.16960159 10.1093/ajcn/84.3.475

[7] McGregor RA, Cameron-Smith D, Poppitt SD. It is not just muscle mass: a review of muscle quality, composition and metabolism during ageing as determinants of muscle function and mobility in later life. Longev Healthspan. 2014;3(1):9. 10.1186/2046-2395-3-9.25520782 10.1186/2046-2395-3-9PMC4268803

[8] Cacciatore S, Calvani R, Esposito I, Massaro C, Gava G, Picca A, et al. Emerging Targets and Treatments for Sarcopenia: A Narrative Review. Nutrients. 2024;16(19):3271. 10.3390/nu16193271.39408239 10.3390/nu16193271PMC11478655

[9] Mesinovic J, McMillan LB, Shore-Lorenti C, De Courten B, Ebeling PR, Scott D. Metabolic Syndrome and Its Associations with Components of Sarcopenia in Overweight and Obese Older Adults. J Clin Med. 2019;8(2):145. 10.3390/jcm8020145.30691198 10.3390/jcm8020145PMC6406767

[10] Mikura K, Kodama E, Iida T, Imai H, Hashizume M, Kigawa Y, et al. Association between sarcopenia and the severity of diabetic polyneuropathy assessed by nerve conduction studies in Japanese patients with type 2 diabetes mellitus. J Diabetes Investig. 2022;13(8):1357–65. 10.1111/jdi.13788.35271762 10.1111/jdi.13788PMC9340862

[11] Yoon JW, Ha YC, Kim KM, Moon JH, Choi SH, Lim S, et al. Hyperglycemia Is Associated with Impaired Muscle Quality in Older Men with Diabetes: The Korean Longitudinal Study on Health and Aging. Diabetes Metab J. 2016;40(2):140–6. 10.4093/dmj.2016.40.2.140.27126884 10.4093/dmj.2016.40.2.140PMC4853221

[12] Kalyani RR, Metter EJ, Egan J, Golden SH, Ferrucci L. Hyperglycemia predicts persistently lower muscle strength with aging. Diabetes Care. 2015;38(1):82–90. 10.2337/dc14-1166.25392294 10.2337/dc14-1166PMC4274779

[13] Baumgartner RN, Wayne SJ, Waters DL, Janssen I, Gallagher D, Morley JE. Sarcopenic obesity predicts instrumental activities of daily living disability in the elderly. Obes Res. 2004;12(12):1995–2004. 10.1038/oby.2004.250.15687401 10.1038/oby.2004.250

[14] van Sloten TT, Savelberg HH, Duimel-Peeters IG, Meijer K, Henry RM, Stehouwer CD, et al. Peripheral neuropathy, decreased muscle strength and obesity are strongly associated with walking in persons with type 2 diabetes without manifest mobility limitations. Diabetes Res Clin Pract. 2011;91(1):32–9. 10.1016/j.diabres.2010.09.030.20965601 10.1016/j.diabres.2010.09.030

[15] Pataky Z, Armand S, Müller-Pinget S, Golay A, Allet L. Effects of obesity on functional capacity. Obesity. 2014;22(1):56–62. 10.1002/oby.20514.23794214 10.1002/oby.20514

[16] Smeuninx B, McKendry J, Wilson D, Martin U, Breen L. Age-Related Anabolic Resistance of Myofibrillar Protein Synthesis Is Exacerbated in Obese Inactive Individuals. J Clin Endocrinol Metab. 2017;102(9):3535–45. 10.1210/jc.2017-00869.28911148 10.1210/jc.2017-00869PMC5587073

[17] Valenzuela PL, Maffiuletti NA, Tringali G, De Col A, Sartorio A. Obesity-associated poor muscle quality: prevalence and association with age, sex, and body mass index. BMC Musculoskelet Disord. 2020;21(1):200. 10.1186/s12891-020-03228-y.32234006 10.1186/s12891-020-03228-yPMC7110672

[18] Andreassen CS, Jakobsen J, Ringgaard S, Ejskjaer N, Andersen H. Accelerated atrophy of lower leg and foot muscles–a follow-up study of long-term diabetic polyneuropathy using magnetic resonance imaging (MRI). Diabetologia. 2009;52(6):1182–91. 10.1007/s00125-009-1320-0.19280173 10.1007/s00125-009-1320-0

[19] Andreassen CSJJ, Andersen H. Muscle weakness: a progressive late complication in diabetic distal symmetric polyneuropathy. Diabetes. 2006;55(3):806–12.16505247 10.2337/diabetes.55.03.06.db05-1237

[20] Tomlinson DJ, Erskine RM, Morse CI, Winwood K, Onambélé-Pearson G. The impact of obesity on skeletal muscle strength and structure through adolescence to old age. Biogerontology. 2016;17(3):467–83. 10.1007/s10522-015-9626-4.26667010 10.1007/s10522-015-9626-4PMC4889641

[21] Carter CS, Justice JN, Thompson L. Lipotoxicity, aging, and muscle contractility: does fiber type matter? Geroscience. 2019;41(3):297–308. 10.1007/s11357-019-00077-z.31227962 10.1007/s11357-019-00077-zPMC6702511

[22] Zoico E, Di Francesco V, Guralnik JM, Mazzali G, Bortolani A, Guariento S, et al. Physical disability and muscular strength in relation to obesity and different body composition indexes in a sample of healthy elderly women. Int J Obes Relat Metab Disord. 2004;28(2):234–41. 10.1038/sj.ijo.0802552.14708033 10.1038/sj.ijo.0802552

[23] Barbat-Artigas S, Rolland Y, Zamboni M, Aubertin-Leheudre M. How to assess functional status: a new muscle quality index. J Nutr Health Aging. 2012;16(1):67–77. 10.1007/s12603-012-0004-5.22238004 10.1007/s12603-012-0004-5PMC12878022

[24] Suetta C, Haddock B, Alcazar J, Noerst T, Hansen OM, Ludvig H, et al. The Copenhagen Sarcopenia Study: lean mass, strength, power, and physical function in a Danish cohort aged 20–93 years. J Cachexia Sarcopenia Muscle. 2019;10(6):1316–29. 10.1002/jcsm.12477.31419087 10.1002/jcsm.12477PMC6903448

[25] Park SW, Goodpaster BH, Strotmeyer ES, de Rekeneire N, Harris TB, Schwartz AV, et al. Decreased muscle strength and quality in older adults with type 2 diabetes: the health, aging, and body composition study. Diabetes. 2006;55(6):1813–8. 10.2337/db05-1183.16731847 10.2337/db05-1183

[26] Park SW, Goodpaster BH, Strotmeyer ES, Kuller LH, Broudeau R, Kammerer C, et al. Accelerated loss of skeletal muscle strength in older adults with type 2 diabetes: the health, aging, and body composition study. Diabetes Care. 2007;30(6):1507–12. 10.2337/dc06-2537.17363749 10.2337/dc06-2537

[27] Yamamoto H, Eshima H, Kakehi S, Kawamori R, Watada H, Tamura Y. Impaired fatigue resistance, sarcoplasmic reticulum function, and mitochondrial activity in soleus muscle of db/db mice. Physiol Rep. 2022;10(18):e15478. 10.14814/phy2.15478.36117307 10.14814/phy2.15478PMC9483406

[28] Allen MD, Major B, Kimpinski K, Doherty TJ, Rice CL. Skeletal muscle morphology and contractile function in relation to muscle denervation in diabetic neuropathy. J Appl Physiol. 2014;116(5):545–52. 10.1152/japplphysiol.01139.2013.24356519 10.1152/japplphysiol.01139.2013PMC3949214

[29] Hesselink MK, Schrauwen-Hinderling V, Schrauwen P. Skeletal muscle mitochondria as a target to prevent or treat type 2 diabetes mellitus. Nat Rev Endocrinol. 2016;12(11):633–45. 10.1038/nrendo.2016.104.27448057 10.1038/nrendo.2016.104

[30] Tallis J, James RS, Seebacher F. The effects of obesity on skeletal muscle contractile function. J Exp Biol. 2018. 10.1242/jeb.163840.29980597 10.1242/jeb.163840

[31] Hilton TNTL, Bohnert KL, Mueller MJ, Sinacore DR. Excessive adipose tissue infiltration in skeletal muscle in individuals with obesity, diabetes mellitus, and peripheral neuropathy: association with performance and function. Phys Ther. 2008;88(11):1336–44.18801853 10.2522/ptj.20080079PMC2579904

[32] Volpato S, Bianchi L, Lauretani F, Lauretani F, Bandinelli S, Guralnik JM, et al. Role of muscle mass and muscle quality in the association between diabetes and gait speed. Diabetes Care. 2012;35(8):1672–9. 10.2337/dc11-2202.22596176 10.2337/dc11-2202PMC3402248

[33] Sacchetti M, Balducci S, Bazzucchi I, Carlucci F, Scotto di Palumbo A, Haxhi J, et al. Neuromuscular dysfunction in diabetes: role of nerve impairment and training status. Med Sci Sports Exerc. 2013;45(1):52–9. 10.1249/MSS.0b013e318269f9bb.22843109 10.1249/MSS.0b013e318269f9bb

[34] Van Eetvelde BLM, Lapauw B, Proot P, Vanden Wyngaert K, Celie B, Cambier D, et al. The impact of sensory and/or sensorimotor neuropathy on lower limb muscle endurance, explosive and maximal muscle strength in patients with type 2 diabetes mellitus. J Diabetes Complications. 2020;34(6):107562. 10.1016/j.jdiacomp.2020.107562.32122790 10.1016/j.jdiacomp.2020.107562

[35] Lafortuna CL, Maffiuletti NA, Agosti F, Sartorio A. Gender variations of body composition, muscle strength and power output in morbid obesity. Int J Obes. 2005;29(7):833–41. 10.1038/sj.ijo.0802955.10.1038/sj.ijo.080295515917862

[36] Dyck PJ, Albers JW, Andersen H, Arezzo JC, Biessels GJ, Bril V, et al. Diabetic polyneuropathies: update on research definition, diagnostic criteria and estimation of severity. Diabetes Metab Res Rev. 2011;27(7):620–8. 10.1002/dmrr.1226.21695763 10.1002/dmrr.1226

[37] Tankisi H, Pugdahl K, Beniczky S, Andersen H, Fuglsang-Frederiksen A. Evidence-based recommendations for examination and diagnostic strategies of polyneuropathy electrodiagnosis. Clin Neurophysiol Pract. 2019;4:214–22. 10.1016/j.cnp.2019.10.005.31886447 10.1016/j.cnp.2019.10.005PMC6921232

[38] Bril VPB. Validation of the Toronto clinical scoring system for diabetic polyneuropathy. Diabetes Care. 2002;25(11):2048–52.12401755 10.2337/diacare.25.11.2048

[39] Wallace TM, Levy JC, Matthews DR. Use and abuse of HOMA modeling. Diabetes Care. 2004;27(6):1487–95. 10.2337/diacare.27.6.1487.15161807 10.2337/diacare.27.6.1487

[40] Pedersen KK, Skovgaard EL, Larsen R, Stengaard M, Sørensen S, Overgaard K. The Applicability of Thigh-Worn vs. Hip-Worn ActiGraph Accelerometers During Walking and Running. J Meas Phys Behav. 2019;2(4):209–17. 10.1123/jmpb.2018-0043.

[41] Montero-Odasso M, van der Velde N, Martin FC, Petrovic M, Tan MP, Ryg J, et al. World guidelines for falls prevention and management for older adults: a global initiative. Age and Ageing. 2022;51(9):afac205. 10.1093/ageing/afac205.36178003 10.1093/ageing/afac205PMC9523684

[42] Andersen H, Jakobsen J. A comparative study of isokinetic dynamometry and manual muscle testing of ankle dorsal and plantar flexors and knee extensors and flexors. Eur Neurol. 1997;37(4):239–42. 10.1159/000117450.9208265 10.1159/000117450

[43] Harbo T, Brincks J, Andersen H. Maximal isokinetic and isometric muscle strength of major muscle groups related to age, body mass, height, and sex in 178 healthy subjects. Eur J Appl Physiol. 2012;112(1):267–75. 10.1007/s00421-011-1975-3.21537927 10.1007/s00421-011-1975-3

[44] Lanza IR, Towse TF, Caldwell GE, Wigmore DM, Kent-Braun JA. Effects of age on human muscle torque, velocity, and power in two muscle groups. J Appl Physiol. 2003;95(6):2361–9. 10.1152/japplphysiol.00724.2002.12923120 10.1152/japplphysiol.00724.2002

[45] Takai Y, Ohta M, Akagi R, Kanehisa H, Kawakami Y, Fukunaga T. Sit-to-stand test to evaluate knee extensor muscle size and strength in the elderly: a novel approach. J Physiol Anthropol. 2009;28(3):123–8. 10.2114/jpa2.28.123.19483373 10.2114/jpa2.28.123

[46] Stouge A, Khan KS, Kristensen AG, Tankisi H, Schlaffke L, Froeling M, et al. MRI of Skeletal Muscles in Participants with Type 2 Diabetes with or without Diabetic Polyneuropathy. Radiol. 2020;297(3):608–19. 10.1148/radiol.2020192647.10.1148/radiol.202019264733048033

[47] Maffiuletti NA, Jubeau M, Munzinger U, Bizzini M, Agosti F, De Col A, et al. Differences in quadriceps muscle strength and fatigue between lean and obese subjects. Eur J Appl Physiol. 2007;101(1):51–9. 10.1007/s00421-007-0471-2.17476522 10.1007/s00421-007-0471-2

[48] Hulens M, Vansant G, Lysens R, Claessens AL, Muls E, Brumagne S. Study of differences in peripheral muscle strength of lean versus obese women: an allometric approach. Int J Obes Relat Metab Disord. 2001;25(5):676–81. 10.1038/sj.ijo.0801560.11360150 10.1038/sj.ijo.0801560

[49] Rolland Y, Lauwers-Cances V, Pahor M, Fillaux J, Grandjean H, Vellas B. Muscle strength in obese elderly women: effect of recreational physical activity in a cross-sectional study. Am J Clin Nutr. 2004;79(4):552–7. 10.1093/ajcn/79.4.552.15051596 10.1093/ajcn/79.4.552

[50] Tomlinson DJ, Erskine RM, Morse CI, Winwood K, Onambélé-Pearson GL. Combined effects of body composition and ageing on joint torque, muscle activation and co-contraction in sedentary women. Age (Dordr). 2014;36(3):9652. 10.1007/s11357-014-9652-1.24744050 10.1007/s11357-014-9652-1PMC4082607

[51] Tomlinson DJ, Erskine RM, Winwood K, Morse CI, Onambélé GL. Obesity decreases both whole muscle and fascicle strength in young females but only exacerbates the aging-related whole muscle level asthenia. Physiol Rep. 2014;2(6):e12030. 10.14814/phy2.12030.24963030 10.14814/phy2.12030PMC4208641

[52] Tomlinson DJ, Erskine RM, Winwood K, Morse CI, Onambélé GL. The impact of obesity on skeletal muscle architecture in untrained young vs old women. J Anat. 2014;225(6):675–84. 10.1111/joa.12248.25315680 10.1111/joa.12248PMC4262351

[53] da Costa Teixeira LA, Soares LA, da Fonseca SF, Gonçalves GT, Dos Santos JM, Viegas ÂA, et al. Analysis of body composition, functionality and muscle-specific strength of older women with obesity, sarcopenia and sarcopenic obesity: a cross-sectional study. Sci Rep. 2024;14(1):24802. 10.1038/s41598-024-76417-7.39438648 10.1038/s41598-024-76417-7PMC11496535

[54] Bosco C, Zanon S, Rusko H, Dal Monte A, Bellotti P, Latteri F, et al. The influence of extra load on the mechanical behavior of skeletal muscle. Eur J Appl Physiol Occup Physiol. 1984;53(2):149–54. 10.1007/bf00422578.6542513 10.1007/BF00422578

[55] Frühbeck G, Busetto L, Dicker D, Yumuk V, Goossens GH, Hebebrand J, et al. The ABCD of Obesity: An EASO Position Statement on a Diagnostic Term with Clinical and Scientific Implications. Obes Facts. 2019;12(2):131–6. 10.1159/000497124.30844811 10.1159/000497124PMC6547280

[56] Wagner R, Heni M, Tabák AG, Machann J, Schick F, Randrianarisoa E, et al. Pathophysiology-based subphenotyping of individuals at elevated risk for type 2 diabetes. Nat Med. 2021;27(1):49–57. 10.1038/s41591-020-1116-9.33398163 10.1038/s41591-020-1116-9

[57] Bays H, Abate N, Chandalia M. Adiposopathy: sick fat causes high blood sugar, high blood pressure and dyslipidemia. Future Cardiol. 2005;1(1):39–59. 10.1517/14796678.1.1.39.19804060 10.1517/14796678.1.1.39

[58] Andersen H, Gadeberg HC, Brock B, Jakobsen J. Muscular atrophy in diabetic neuropathy: a stereological magnetic resonance imaging study. Diabetologia. 1997;40:1062–9.9300243 10.1007/s001250050788

[59] Krssak M, Falk Petersen K, Dresner A, DiPietro L, Vogel SM, Rothman DL, et al. Intramyocellular lipid concentrations are correlated with insulin sensitivity in humans: a 1H NMR spectroscopy study. Diabetologia. 1999;42(1):113–6. 10.1007/s001250051123.10027589 10.1007/s001250051123

[60] Reiter DA, Bellissimo MP, Zhou L, Boebinger S, Wells GD, Jones DP, et al. Increased adiposity is associated with altered skeletal muscle energetics. J Appl Physiol. 2023;134(5):1083–92. 10.1152/japplphysiol.00387.2022.36759162 10.1152/japplphysiol.00387.2022PMC10125027

[61] Schaap LA, Pluijm SM, Deeg DJ, Visser M. Inflammatory markers and loss of muscle mass (sarcopenia) and strength. Am J Med. 2006;119(6):526.e9-17. 10.1016/j.amjmed.2005.10.049.16750969 10.1016/j.amjmed.2005.10.049

[62] Favretto MA, Cossul S, Andreis FR, Marques JLB, editors. Evaluation of Rate of Muscular Force Development in Type 2 Diabetic Individuals with and without Diabetic Peripheral Neuropathy. XXVI Brazilian Congress on Biomedical Engineering; 2019 2019//; Singapore: Springer Singapore.

[63] Jakobsson F, Borg K, Edström L, Grimby L. Use of motor units in relation to muscle fiber type and size in man. Muscle Nerve. 1988;11(12):1211–8. 10.1002/mus.880111205.3070382 10.1002/mus.880111205

[64] Larsson L, Grimby G, Karlsson J. Muscle strength and speed of movement in relation to age and muscle morphology. J Appl Physiol Respir Environ Exerc Physiol. 1979;46(3):451–6. 10.1152/jappl.1979.46.3.451.438011 10.1152/jappl.1979.46.3.451

[65] Fisher G, Windham ST, Griffin P, Warren JL, Gower BA, Hunter GR. Associations of human skeletal muscle fiber type and insulin sensitivity, blood lipids, and vascular hemodynamics in a cohort of premenopausal women. Eur J Appl Physiol. 2017;117(7):1413–22. 10.1007/s00421-017-3634-9.28497385 10.1007/s00421-017-3634-9PMC5963254

[66] Farup J, Just J, de Paoli F, Lin L, Jensen JB, Billeskov T, et al. Human skeletal muscle CD90(+) fibro-adipogenic progenitors are associated with muscle degeneration in type 2 diabetic patients. Cell Metab. 2021;33(11):2201-14.e11. 10.1016/j.cmet.2021.10.001.34678202 10.1016/j.cmet.2021.10.001PMC9165662

[67] Cuoco A, Callahan DM, Sayers S, Frontera WR, Bean J, Fielding RA. Impact of muscle power and force on gait speed in disabled older men and women. J Gerontol A Biol Sci Med Sci. 2004;59(11):1200–6. 10.1093/gerona/59.11.1200.15602076 10.1093/gerona/59.11.1200

